# A Two-Stage DEA Model to Evaluate the Technical Eco-Efficiency Indicator in the EU Countries

**DOI:** 10.3390/ijerph18063038

**Published:** 2021-03-16

**Authors:** Victor Moutinho, Mara Madaleno

**Affiliations:** 1 Management and Economics Department and NECE-UBI, University of Beira Interior, Rua Marquês d’Ávila e Bolama, 6201-001 Covilhã, Portugal; 2 GOVCOPP—Research Unit in Governance, Competitiveness and Public Policy, Department of Economics, Management, Industrial Engineering and Tourism (DEGEIT), University of Aveiro, 3810-193 Aveiro, Portugal; maramadaleno@ua.pt

**Keywords:** air pollutants, data envelopment analysis, eco-efficiency, fractional regression models, GHG emissions

## Abstract

This paper evaluates the evolution of eco-efficiency for the 27 European Union (EU) countries over the period 2008–2018, provided the traditional high concerns of the EU concerning the economic growth-environmental performance relationship. The EU has triggered several initiatives and regulations regarding environmental protection over the years, but as well the Sustainable Development Goals demand it. Under this setting, we conduct a two-stage analysis, which computes eco-efficiency scores in the first stage for each of the pairs EU 27-year, through the nonparametric method data envelopment analysis (DEA), considering the ratio GDP per capita and greenhouse gas emissions (GHG). In the second stage, scores are used as a dependent variable in the proposed fractional regression model (FRM), whose determinants considered were eight pollutants (three greenhouse gases and five atmospheric pollutants). CO_2_/area and N_2_O/area effects are negative and significant, improving the eco-efficiency of the EU 27 countries. When the efficient European countries are excluded from the estimations, the results evidence that CO_2_/area and CH_4_/area decrease the DEA score. The country with the lowest GHG emissions and pollutant gases was Ireland, being the country within the considered period that mostly reduced emissions, particularly SOx and PM10, increasing its score.

## 1. Introduction

Over time, the European Union (EU) has concentrated great efforts and commitments on reducing environmental pollution. Addressing the issue of GHG emissions leads to the Kyoto Protocol. Regarding the emissions of polluting gases, the EU countries approved limitations in 2016. Here, limits are placed on sulfur dioxide (SO2), nitrogen oxides (NOX), volatile organic compounds except for methane (NMOVCs), ammonia (NH3), and particles with a diameter of less than 2.5 µm (PM2.5) [1]. Total emissions from human activities of sulfur oxides (SOx) and nitrogen oxides (NOx) are given as quantities of SO_2_ and NO_2_. Recently, the 2030 climate targets plan can be mentioned, where the European Commission (EC) proposes a reduction of GHG emissions by at least 55%. This proposal aims to achieve climate neutrality by 2050, in line with the Paris Agreement [2], to keep the global temperature rise below 2 °C (not to exceed 1.5 °C) [3]. As established, climate change is a topic of increasing concern among lawmakers and different generations. Therefore, this work comes with the motivation to add information that contributes to the formulation of more anchored environmental measures. To this end, the study of eco-efficiency was used [4]. This translates into a ratio that links the economic value of goods and services with the environmental impacts associated with their production, where the higher the value of the ratio, the better the eco-efficiency [5]. Generally, this ratio appears with CO_2_ to be divided by GDP, presenting the problem of not translating the reality of production, since this is not just the only polluting gas emitted, turning existent analysis limited to account for the entire problem.

Many authors have made important contributions to this theme of eco-efficiency, as we will see in the literature review section, which shaped the thinking of several researchers until a concrete relationship was reached, measured by the ratio between economic growth and greenhouse gas emissions or pollutants (GHG). In the European context, several efforts have been made to reduce GHG and Pollutant Gas emissions since this is a theme, not only with an environmental impact but also with an economic and social impact [6]. Thus, this theme (of emissions reduction) started with the Quito Protocol (discussed in 1997, opened for signatures in 1998, and ratified in 1999, entering into full operation on 16 February 2005). According to this document, developed countries had to impose a reduction of GHGs by at least 5.2% to 1990 levels in the period 2008 and 2012 (also known as “first commitment period”) [7]. To comply with the imposed obligations, the EU developed a measurement system for GHG emissions, as well as implemented a trading system for emissions licenses [6]. For what is considered the “second commitment period”, 2013 to 2020, the countries participating in the Kyoto agreement agreed to a 20% reduction compared to the base year (1990). In 2015, the Paris Agreement was signed, where 195 countries pledged to keep the average global temperature below 2 °C. To contribute to this goal, the EU adopted new targets in the environmental and energy areas by 2030, namely a reduction of GHG emissions of at least 40% (compared to the base year) [8,9]. Regarding limitations on polluting gases, a directive with targets for each European country between 2020 and 2029 and for years after 2030 was approved in 2016 [10]. In addition to these commitments, the European Commission presented in 2019, at the COP25 Climate Summit, in Madrid, the “European Green Deal”. By 2050, the EU must become climate neutral, and by 2030, CO_2_ emissions must be reduced by 50% (compared to 1990 levels) [6].

The eco-efficiency indicator has become an increasingly consistent tool to assist decision-making and provide better economic, environmental, and social performance [11]. This indicator was introduced around 1990 by the World Business Council for Sustainable Development (WBCSD), which defines it as “The New Eco-efficiency analysis considered structural Greenhouse and Air pollutions emissions by area: A case study from Europe 7 delivery of competitively priced goods and services that satisfy human needs and bring the quality of life, while progressively reducing ecological impacts and resource intensity throughout the life-cycle to a level at least in line with the Earth’s estimated carrying capacity” [12]. In practice, eco-efficiency translates into the measurement of a ratio between the economic value of the goods and the environmental impacts involved with the production processes, and the higher the ratio, the better the eco-efficiency [5]. Regarding GHG emissions, the most usual is to use a ratio between GDP and CO_2_. However, this analysis has limitations as it does not include the production process that involves several GHG emissions and that a given GDP can be obtained with different combinations of polluting gases [5]. Thus, taking into account these limitations, [13,14] developed very interesting works in an attempt to overcome them. These authors analyzed global eco-efficiency by calculating a composite country-level ecological performance indicator for 20 EU members. According to [15], it is necessary to reinforce the difference between eco-efficiency and concepts, such as sustainability (see [13]), environmental effectiveness (see [16]), or environmental performance indicators, even if the differences between them are tenuous.

This investigation differs from the previous ones when studying the eco-efficiency measured by the ratio between the GDPpc and the GHG emissions by area for all the member states of Europe (EU-27), proceeding afterward with a comparison of the eco-efficiency estimated by the nonparametric method data envelopment analysis (DEA), in which we consider the ratio between economic growth per capita and greenhouse gas emissions to be maximized, and as inputs the classics, namely, the production inputs capital per capita and labor per capita, energy use, electricity produced, and deviation of average near-surface temperature (see Table 1a for more details). Afterward, in the second step of our econometric approach, the eco-efficiency scores are used as a dependent variable in the proposed fractional regression model (FRM) econometric relationship whose determinants considered were the eight pollutants, namely three greenhouse gases and five atmospheric pollutants (see Table 1b for more details).

The rest of this paper is organized as follows: Section 2 presents a brief state-of-the-art regarding the eco-efficiency literature in the EU countries. Section 3 shows the data used and the methodology applied. Section 4 is dedicated to revealing the main results of this paper, and their discussion is displayed in Section 5 along with policy implications. Lastly, Section 6 offers the main conclusions.

## 2. Brief and Recent Literature Review

### 2.1. Framework

Recent and innovative eco-efficiency analysis is done with adjusted production models, where a production frontier is used to infer the inputs and outputs relationship. Here, pollutant emissions might be observed as undesirable inputs and/or outputs. Bu determining the efficiency boundary, the research explores the link between economic and environmental results. The goal is to derive environmental and economic growth efficiency measures [13,14,17,18,19,20,21,22]. Previous literature mentions that eco-efficiency measures are related to the economic output value obtained through the production process, considering existent environmental pressures from this production. Recent interest in estimating eco-efficiency through technical analysis emerged [23,24,25,26,27,28,29,30,31]. However, also recently, a content analysis study reveals that eco-efficiency has lost significance in favor of the circular economy discourse in the EU research funding programs of Horizon 2020, at least since the introduction of the 2011 Eco-Innovation Action Plan [32]. Still, ref. [33] mentions that eco-efficiency indicators represent a valuable instrument for policy-making and decisions undertaken geared at sustainability. They call attention to the need to focus on heterogeneous territorial settings and specific economic structures.

As argued previously, eco-efficiency scores computation through DEA methods is relatively recent. Ref. [34] apply a two-stage network DEA to OECD countries to find that Switzerland is highest in eco-efficiency (measured using the GDP/GHG ratio) and Estonia in eco-innovation. Combining multi-regional environmentally extended input–output tables, and DEA, Ref. [35] quantified the eco-efficiency of 14 manufacturing sectors in the EU. They identified the sectors and pollutants requiring more stringent regulations and pollutants requiring more stringent regulations and where to perform higher investments in cleaner technologies. Considering, as we do, the EU 27 countries, [36] model eco-efficiency performance using a metafrontier framework for 14 manufacturing industries. They use NOx, SOx, CO_2_, CH_4_, N_2_O, CO, NMVOC, and NH_3_ as undesirable outputs to represent the impacts these industries cause in the environment. Conclusions indicate that heavy industries perform eco-inefficiently. For the electricity sector, ref. [37] explore eco-efficiency in the EU 28 countries combining DEA analysis output with input–output analysis. In their DEA analysis, the output considered was gross-value added, and inputs were labor, capital stock, GHG emissions, acidifying gas missions, and ozone precursors.

Furthermore, relying upon the metafrontier DEA analysis, ref. [33] tried to compare the evolution of eco-efficiency in 282 European regions and to highlight the technology and conditional efficiency gaps they reveal. Results confirm the overall upward trend in eco-efficiency but no evidence of regional convergence. In their DEA model, GDP pc is used as output and the employment rate and domestic material consumption per capita as inputs. Furthermore, from a regional perspective, this time for cities, ref. [31] analyze the effects of urban air pollution in 24 German cities using DEA and stochastic frontier analysis (SFA) to compute eco-efficiency scores. In the second stage, a fractional regression is used to find that excess PM10, average temperature, average NO_2_ concentration, and rainfall impact significantly eco-efficiency (measured by the GDP/CO_2_ emissions ratio). [38], as well, use DEA to derive eco-efficiency ratios and afterward use a linear regression model to find that in the short and long-run, roof insulation thickness and material followed by exterior wall insulation material are the factors that most influenced these ratios.

Eco-efficiency represents the efficiency of resources used [29]. [29] assessed through the slacks-based measure DEA model the eco-efficiency for 17 European countries concluding that more efforts are needed to its improvement. Two outputs are used (the desired GDP pc and the undesired CO2 emissions pc), while energy consumption, labor productivity, the share of renewable energy in total energy consumption, and gross capital formation productivity are used as inputs. In the same year, ref. [39] aimed to assess environmental efficiencies resulting from waste generation in 15 European countries, where eco-efficiency has been measured through the GDP/GHG emissions per capita ratio and using the DEA methodology. Previously, ref. [15] evaluate the environmental performance of the EU through a two-stage analysis. First, the authors develop environmental performance indicators for three air pollutants (CO2e, SO2, NOx) using DEA. Second, a model of explicit distribution dynamics is proposed. Still, abatement opportunities were found to be still remarkable even after analyzing 18 years of data in 27 countries. Furthermore, [30] used GDP over CO2 emissions to account for eco-efficiency in German cities through DEA and the Malmquist productivity indexes also used by [29].

### 2.2. Hypothesis, Motivation and Contribution Details

Regarding the previous literature review performed, in terms of the novelty of the article, we will say that our research focuses on a first step in assessing technical efficiency at the economic and environmental level, which will be defined by the ratio between the measure of economic growth, GDP per capita, given the different dimension in terms of marginal contribution of each country to mitigate greenhouse gas emissions by geographic density (per km^2^). This metric will be the proposed output to be considered at the frontier of technical efficiency defined in a nonparametric relationship, using DEA, since it is possible to analyze and evaluate (i) the effect of the selected inputs to estimate the nonparametric relationship, namely the most commonly used variables, referred to in the capital and labor literature (economic variables that influence GDP), together with the energy variables, energy used and electricity generation that influence gas emissions, that is, simultaneously influence the proposed eco-efficiency measure. It is as well highlighted that we do not estimate panel data, but eco-efficiency scores are estimated through times series cross-sections [40]; (ii) the inclusion of these inputs weighted by the geographical area allows us to take into account the effects of the geographical dimension and which influence eco-efficiency as well [41,42]; (iii) the effect of the selected inputs in the variation of economic and environmental inefficiency, taking into account that the EU 27 sample includes older economies in terms of EU membership and the most recent ones in EU membership, whose rules and consolidated energy and environmental policy commitments in terms of European regulation and regulation can explain the different scoring of eco-efficiency resulting from the DEA [43]; (iv) besides commonly used inputs by the literature, none of the previous studies of eco-efficiency accounted for the development of deviations in average near-surface temperature by country as an input. We do that considering the fact that countries have the Kyoto compromises assumed with respect to decreasing global earth temperature, and as well these deviations will be important to explain countries’ heterogeneous eco-efficiency scores. In a second step, in terms of the novelty of the article [44,45] (v), a parametric relationship is included in the analysis, the gases and pollutants taking into account their structure, as explanatory determinants and considered as control variables that affect the eco-efficiency explained in the efficiency scores that are translated into a percentage scale in order to consider the percentage values on a scale of zero 0% to 100%, whereas 100% corresponds to the maximum relative efficiency value and which corresponds to the value 1 on the technical efficiency frontier. It should also be noted that the choice of these regressors, the greenhouse gases in the inclusion of the border regression, as well as the inclusion of the economic and geographical weightings were taken into account, constituting the main contribution of the present article to the existent literature.

In terms of identified gaps, the comprehensive review of the existent literature on eco-efficiency has made it possible to identify that for European Countries; there is a gap in the DEA analysis of economic and environmental efficiency for this set of countries/economies considering, on one hand, the economic weights per capita and the emission of greenhouse gases by geographic area. On the other hand, there are few studies that consider in a 2nd stage in the analysis of eco-efficiency the influence of pollutant determinants at the level of the stochastic boundaries, and consequently affect the eco-efficiency, making it possible to identify the significance and magnitude of the greatest positive or negative impact on different levels of eco-efficiency within the panel of European Economies. Two-steps estimation has as well been presented by [43,44], although considering different influencing variables.

Therefore, two main hypotheses were raised here, and to explore them, a parametric relationship is included in the analysis. First, we wanted to test the hypothesis that different gases exert different effects over eco-efficiency scores (3 greenhouse gases); Second, the hypothesis that different pollutants (5 atmospheric pollutants), taking into account their structure as explanatory determinants, will differently affect the eco-efficiency explained in the efficiency scores computed in the first part of our methodology. In fact, as well for the EU 27 countries, ref. [36] model eco-efficiency performance using a metafrontier framework for 14 manufacturing industries, using NOx, SOx, CO_2_, CH_4_, N_2_O, CO, NMVOC, and NH_3_ as undesirable outputs to represent the impacts these industries cause in the environment, finding that heavy industries perform eco-inefficiently.

Although European countries are more aware of pollution and environmental issues, sustainable development reached up to now is not anticipated and desired. Several studies explore the convergence of undesirable outputs but mainly do it considering CO_2_ [27,33,34,37]. This is a common feature, and, traditionally, simple eco-efficiency ratios are used based on carbon solely [36]. Nonetheless, ignoring gases and other pollutants as part of the eco-efficiency process is very limiting. [36] argue they can be considered as partial indexes of eco-efficiency. Still, the authors considered different environmental indicators in their eco-efficiency estimated ratios. In the present study, we consider general GHG emissions by area in the calculous of eco-efficiency scores computed cross-sectionally [41] but argue that these different pollutants will have different impacts on these eco-efficiency scores by country. To test our hypothesis in the second part of the methodology, as already stated, we use the FRM methodology, and clear differences are highlighted. This is done considering that the quantity of GDP produced is not the most relevant under the new demanding environmental and sustainable demanding scenarios imposed through EU legislation and whose policymakers need to face. The most important now is to consider that the production process involves several GHG emissions, and the quality of the GDP may be the result of different combinations of polluting gases [5,13,14,15,16,36,45].

Furthermore, ref. [46] evaluated environmental performance in the EU through DEA during 2000–2017 and the global Malmquist–Luenberger index [46]. Similar to [36] the authors’ main contribution was to consider different types of undesirable outputs and using long-term panel data on EU countries. Our study differs from these of both authors [36,47] considering as output the GDP/GHG ratio, as previously mentioned, and by examining in a second stage the impact of both gases and pollutants over the eco-efficiency score computed cross-sectionally during 2008–2018. Moreover, ref. [45] follows [46] and only includes CO_2_, PM_2.5_ and waste emissions as outputs and uses panel data analysis. Our selection of gases and pollutants included as explanatory variables of DEA computed eco-efficiency scores rely on their significant and heterogeneous impact on countries’ environmental efficiency. In the present article, we do not use the Malmquist index (MPI) as [46,47] to assess eco-efficiency, as in the studies that served as the basis for the DEA two-stage method, as it was not our concern to analyze eco-efficiency intertemporal productivity performance in EU-27, in the 2008–2018 period. Therefore, we were not concerned with explaining the inducing factors that are associated with changes in technical efficiency and changes in technology efficiency, but only to analyze the eco-efficiency rankings evidenced each year for the cross-section of European countries, and to understand if in the time horizon considered there were changes in positioning in each of the sample’s European countries, to afterward evaluate, which factors affect these rankings the most (gases or pollutants). Thus, in the perspective of static evaluation, globally, cross-section DEA models are used for a given period of time, as referred to in previous studies [27,43,45], among others.

## 3. Data and Methodology

### 3.1. Data

In this study, we use annual data from the 2008–2018 period, after the Kyoto Commitment protocol signature, using the panel of 27 EU countries, considering the disposable data. Two distinct groups of these EU 27 countries were considered, namely the EU 15 (Belgium, Denmark, Germany, Greece, Finland, France, Greece, Hungary, Ireland, Italy, Luxembourg, Netherlands, Poland, Portugal, Spain, Sweden, and United Kingdom) and the EU 12 (Bulgaria, Cyprus, Czech Republic, Estonia, Hungary, Lithuania, Latvia, Malta, Poland, Romania, Slovenia, and Slovakia). Variables presented in Table 1a were those included in the first econometric specification of the study, where the efficiency frontier under the output orientation was computed. The output variable used was the ratio GDP pc/GHG/area, which has been maximized for the verified inputs (Table 1a) by using the DEA method. The values of the efficiency frontier were calculated by assuming both constant returns to scale (CRS) and variable returns to scale (VRS).

The ratio between the value of gross domestic product per capita and the value of the volume of GHG emissions by area are considered as outputs, and the total Labor force per capita, gross capital formation per capita, primary energy used before transformation to other end-use fuels, electricity generated by fossil fuels included oil. Gas, coal, and derived fuels and the deviations in average near-surface temperature are considered as inputs. All the variables, except deviations in average near-surface temperature, were converted into their natural logarithm.

The use of output and input variables is based on the literature and adapted in accordance. For the first stage, and considering the GDP/GHG ratio (output), this one was used following [21,22,27,29,30,31,39], among others. The inclusion of the inputs labor force and capital formation relies on the traditional production function [30,37,48,49,50,51]. The inclusion of energy use and electricity generated through fossil fuels to determine the eco-efficiency scores is because both are considered factors that strongly influence economic growth and GHG emissions [29,37]. Finally, the input deviation in average near-surface temperature is included based on the study developed by [51].

Table 1b presents the variables used in the second stage of our econometric application, the application of the fractional regression models (FRM). In this model, the dependent variable was the eco-efficiency scores obtained from the first stage, namely the scores reached by year and country through DEA (values of the scores of technical eco-efficiency according to DEA technique under the VRS assumption). Besides presenting data and sources, Table 1a,b presents data descriptive statistics.

Carbon dioxide emissions (CO_2_), methane emissions (CH_4_), nitrous oxide emissions (N_2_O), ammonia (NH_3_), nonmethane volatile organic compounds (NMVOC), fine particulates PM2.5 and PM10, and sulfur oxide emissions (SO_x_) were added as explanatory variables. The idea is to infer how emissions of different pollutants affect the EU 27 eco-efficiency scores through time, to observe both impacts, significance and evolution throughout the years.

Therefore, in the second stage of our econometric development, the eco-efficiency scores for European countries obtained from the DEA model (output maximized GDP/GHG) were explained by a set of explanatory variables, all related to the emission of pollutants where we have included eight emitted substances’ to the atmosphere. To be exact, we considered the effects of the three main greenhouse gases CO_2_, CH_4_, N_2_O, and five air pollutants—PM10, PM2.5, NMVOC, NH_3_, SOx.

All these pollutants were as well considered by [52] in their study. According to [53] cited by [51], the total emission of pollutants into the atmosphere derives from nine sectors, namely energy production and distribution, energy use in industry, road transport, non-road transport, agriculture, commercial, institutional and households, industrial processes and product use, waste, and other sources (see [52] for more details). Table A1 in the Appendix A provided information about the correlation of inputs and the output ratio for the DEA scores.

### 3.2. Methodology

#### 3.2.1. Data Envelopment Analysis (DEA)

Mathematical linear programming is behind the DEA method, which is simply a decision-making tool used to measure the relative productive efficiencies between comparable decision-making units (DMUs). It has been proposed by [53], is a nonparametric technique that estimates production frontiers and evaluates the efficiency of DMUs (comparable units using the same resources at different proportions), which in this article refers to each of the EU 27 countries. The period considered was 2008–2018 for all the 27 countries in the European Union (11 × 27 = 297 DMUs). Variables and periods of analysis were selected based on the literature review, data availability, and their previously reported significant impact on countries’ environmental and eco-efficiency.

The DEA model does not impose an initial explicit functional form, not requiring prior assignments of inputs and outputs weights [54]. It simply determines an envelopment surface named the empirical production function or easier the efficient frontier. By not imposing weights, these are derived from the data during the DEA application, changing among DMUs. The production possibility set is thus represented by the efficient frontier being the set of all possible combinations between the inputs and outputs as in a production process, bounding the DMUs area. To maximize the relative efficiency (RE) of unit *i*_0_ or adopt the most favorable set of weights for DMUs implies use Equation (1):

(1) $$RE_{i_{0}}=max\frac{\sum_{p=1}^{s}v_{p}y_{pi_{0}}}{\sum_{q=1}^{m}w_{q}x_{pi_{0}}}$$

subject to: $\frac{\sum_{p=1}^{s}v_{p}y_{pi}}{\sum_{q=1}^{m}w_{q}x_{pi}}\;\leq 1,\;i=1,\;\ldots ,\;n;\;v_{p}\;\geq \;\varepsilon ,\;p\;=\;1,\;2,\;3,\;\ldots ,\;s;\;w_{q}\;\geq \;\varepsilon ,\;q\;=\;1,\;2,\;3,\;\ldots ,\;m$. Thus, we have a function that corresponds to a ratio of the weighted sum of the outputs regarding the weighted sum of the inputs. The weights for each DMU are established by the DEA model, being $RE_{i_{0}}$ the score of the relative efficiency of the unit *i*_0_ or DMUi_0_; *y* and *x* are, respectively, the outputs and inputs, with weights *v* and *w*. *p* is the number of outputs (*p* = 1, 2, 3, …, *s*); *q* is the number of inputs (*q* = 1, 2, 3, …, *m*), whereas n is the number of DMUs of the sample.

Regarding efficiency scores to be computed, the most efficient DMUs (EU 27 countries) are those obtaining the best combination between inputs and outputs (if the efficiency ratio is one, the country is the most efficient; lower than one, being considered inefficient), considered afterward as benchmarks for the most inefficient DMUs. Within the present context, being efficient relates to the ability to reduce GHG and increase GDP simultaneously during the production process. Therefore, a country will be efficient by reaching the maximum output level with the available inputs or by using the lowest amount of inputs for a given output level. In the present specification, European efficiency is computed through the DEA model considering the output orientation (constant inputs and higher GDP/GHG output). With the output orientation, we need to linearize Equation (1), and for this, the denominator must be minimized, and the numerator must equal one (Equation (2)).

(2) $$RE_{i_{0}}=min\sum_{q=1}^{m}w_{q}x_{pi_{0}}$$

Subject to: $\sum_{p=1}^{s}v_{p}y_{pi_{0}}=1$; $\sum_{p=1}^{s}v_{p}y_{pi}-\;\sum_{q=1}^{m}w_{q}x_{pi}\;\leq 0$; $v_{p}\;\geq \;\varepsilon ,\;p\;=\;1,\;2,\;3,\;\ldots ,\;s$; $w_{q}\;\geq \;\varepsilon ,\;q\;=\;1,\;2,\;3,\;\ldots ,\;m$. In the output-oriented DEA model, the obtained scores reveal the output amount that could be increased using the same inputs to reach the 100% efficiency level, leading that the ratio of the weighted sum of outputs over the weighted sum of inputs should be equal to 1. By assuming constant returns to scale (CRS), the $RE_{i_{0}}{}^{*}$ is the optimal efficiency score for the DMUi_0_, being ε an infinitesimal positive number. Both the DEA model Charnes–Cooper–Rhodes (CCR) of [54] assuming CRS and the DEA model assuming variable returns to scale (VRS) are used, provided the envelopment surface will differ depending on the scale assumptions (CRS or VRS) that underpin the model. While VRS encompasses both increasing, constant, and decreasing returns to scale, reflecting the production technology behavior, the CRS reflects the fact that output will change by the same proportion as inputs are changed. The DMUs are compared with all the DMU’s of the sample and evaluated considering the performance of others, allowing to compute global technical efficiency measures.

#### 3.2.2. Fractional Regression Model (FRM)

In our eco-efficiency analysis, the second step corresponds to using the obtained DEA scores from the first step and analyze its relationship with possible influencing factors, namely eight different types of pollutants, using the FRM approach. The methodology is deeply explored in [55], where the authors considered four binomial models, namely the logit, probit, log-log, and complementary log-log (cloglog) for the one part and second part models. By using the FRM model, usual econometric problems associated with the linear models’ applications to DEA scores are surpassed. The model was proposed by [56] and used in the literature as the most appropriate to be applied when the dependent variable (y) results from DEA scores [57,58] since it is required for the dependent variable to be within the interval [0, 1] ([56]). The FRM model enforces the desired constraints of the conditional mean of y, independently of its functional form. As well, E(y│x) = G(xθ) (the distribution of y conditional on x) is bounded to that same interval, being G(.) a nonlinear function satisfying 0 ≤ G(.) ≤ 1. Moreover, [57] suggest the FRM estimation by using the quasi maximum-likelihood (QML) based in the Bernoulli log-likelihood function ($LL_{i}\;\left(\theta \right)=y_{i}log\left[G\left(x_{i}\theta \right)\right]+\left(1-y_{i}\right)log\left[1-G\left(x_{i}\theta \right)\right]$). Considering that E(y│x) is correctly specified, the θ QML estimator is consistent and asymptotically normal [56]. Therefore, any cumulative distribution function applied to model binary data is possible [56].

In one-part models, one assumes that variables will exert the same effect across efficient and non-efficient DMUs, but since this may not happen always, we should apply the two-part models [56]. Concerning the two-part models, in the first component, we analyze the probability of observing an efficient DMU, where each DMU is turned into a binary variable that assumes the value 0 when 0 < y < 1 (inefficient DMU) and 1 when y = 1 (efficient DMU). The conditional probability is estimated through the maximum-likelihood method. In the second component of the estimation of the two-part models, efficient countries are removed from estimations. The goal is that in the two-part models, the change in the scores of inefficient DMUs weighted by their observational probability is computed in the first component, and the change in the probability of observing an efficient DMU weighted by one minus the expected score of an inefficient DMU is computed in the second component [56].

Succeeding [56], we employed the RESET test able to detect possible misspecifications of the functional form of the conditional mean. This test is applied to assume that in the null hypothesis, the model follows a correct specification. As well, the *P*-test was computed [57], which tests the alternative FRM one-part and two-part models. Moreover, we have also applied the GOFF-I and GOFF-II tests to infer about the goodness-of-functional form and the generalized goodness-of-functional form (GGOFF) test.

## 4. Results

### 4.1. First Stage: DEA Method

Table 2 and Table 3 display the results of the DEA model of the eco-efficiency scores obtained for the 27 European countries, considering constant returns to scale and variable returns to scale, respectively. To further explore the scores obtained from the DEA method, both tables show the evolution of the DEA score by country and year in the 2008–2018 period analyzed.

It is important to highlight that almost all countries show a growing trend for their score. Mainly, the countries that present a higher average of their score show a positive evolution of the eco-efficiency score. Hence, for instance, the results of the DEA model under the CRS assumption reveal the top five average scoring values in Sweden, Hungary, Czech Republic, France, and Spain (Table 2). Regarding the DEA model under the VRS assumption (Table 3), the results show that the top five average scoring values representing the maximum eco-efficiency are achieved by Hungary, France, Spain, Italy, and Sweden, for the entire period. Therefore, results are very similar in what concerns the top five, differing only in order and in one country. Results also imply that in both assumptions (constant and variable return to scale), countries in the mentioned top ranking are on the eco-efficiency frontier and are considered efficient, while all the other countries are considered as Eco-inefficient. Oppositely, the three lowest eco-efficiency scores are attributed to Luxembourg, Malta, and the Netherlands in both DEA-VRS and DEA-CRS specifications.

It is also important to highlight that when the eco-efficiency levels are considered on average in the European Group, the technical efficiency is far from being achieved by the 27 EU countries. This result is more evident under the DEA-CRS specification, whereas under the DEA-VRS model, the reported values are always in the interval (94–97%), favoring the VRS specification under the econometric specifications.

Considering that this paper performs an output-oriented estimation considering separately the EU 15 group and the EU 12 group, in accordance with the former and last integration of these countries in the European Community, respectively, results achieved imply that during the 2008–2018 period, for instance, in the year 2010, the DEA-CRS model evidence that in the countries for EU 15, namely Sweden, France, Italy, Spain Portugal, and United Kingdom, we have the maximum 100% value of frontier. However, for the EU 12 group, Hungary, Malta, Poland, Romania, and Slovakia, results of eco-efficiency scores reveal the same 100% value of frontier of technical eco-efficiency levels for these countries.

As well, average values for the entire period are always higher under the DEA-VRS specification, indicating that when encompassing both increasing, constant, and decreasing returns to scale, reflecting the production technology behavior, eco-efficiency scores are higher. Provided that the CRS reflects the fact that output will change by the same proportion as inputs are changed solely, results on average for the entire period, by country, and by year as well are tendentially lower. Considering these results, we will present in the next section the FRM model results considering the DEA-VRS scores.

### 4.2. Second Stage: Fractional Regression Model

In the empirical application, the results of the FRM consider as the dependent variable the eco-efficiency scores based on the DEA-VRS technique or the scores of the pure technical efficiency (PTE). The results of the specification tests for the one-part models and both of the two-part models are presented in Table 4. Regarding the logit, probit, log-log, and cloglog one-part model specifications, according to the results of the RESET test, GOFF I, GOFF II, and GGOFF tests, the statistical evidence (at 1% and 5% level) reject all the null hypothesis, except the cloglog specification proving that the current specification is adequate. This same statistical evidence is sustained regarding the values and p-values showed in the *P*-test, where each model specification is tested against all the others. In this case, the cloglog specification is always accepted. This result implies that the *P*-test does not provide an unequivocal conclusion, and therefore, in the rest of this paper, we present only the cloglog specification, which was simultaneously supported by the RESET, GOFF I, GOFF II, and GGOFF tests [59].

Concerning the first component of the two-part models, with statistically significant evidence (at 1% and 5% level) on the RESET test, GOFF I, GOFF II, and GGOFF tests, we will reject all the null hypotheses proving that logit and cloglog are the adequate specifications. However, when specifications are tested against other specifications (in the *P*-test), results presented in Table 4 confirm that the logit and loglog specifications are never rejected. For this reason, for these two-part models, these specifications are considered more suitable. Moreover, for the second component of the two-part models, when the *P*-test is also analyzed, except that cloglog is never rejected, which supports its suitability for this estimation. In sum, the FRM was only estimated for suitable specifications [59,60]. Thus, for the one-part model, the estimations are exposed by using logit and cloglog specifications; for the first component of the two-part model, the estimations are displayed, and for the second component of the two-part model, the cloglog specification is shown.

The results for all the four specifications of FRM are disclosed in Table 5. Results from the one-part model, under the cloglog specification, show that two GHG emissions, more specific CO_2_/area and N_2_O/area, are negative and significant at the 1% level, lowering the eco-efficiency of the EU 27 countries. However, when the first component of the two-part model is analyzed, according to the logit and cloglog specifications, we can observe that these same two types of GHG emissions exert negative and statistically significant pressure on eco-efficiency scores at 5% and 10%, respectively. In the second component, when the efficient European countries are excluded from the estimations, the results presented in Table 5 show that all pollutants decrease the DEA score with significance in the EU 27 panel, although NMVOC is not statistically significant under the loglog specification.

The existence of the three GHG emissions decreases the eco-efficiency of the European countries in terms of economic growth, leading to higher environmental pressures in both one-part and two-part models. Additionally, the coefficients of other pollutants, PM2.5/area, PM10/area, and NH3/area, are negative and significant at 1%, which is aligned with what was theoretically expected in both the one-part model under the cloglog specification and according to the logit and cloglog specifications from the first component of the two-part model. As well, PM2.5/area, PM10/area, and SOx/area are negative and significant at 1%, which is aligned with theoretical expectations in the two-part model second component, regarding the cloglog specification. In that same specification in the two-part model second component, the explanatory variables regarding the pollutants NH_3_/area and NMVOC/area are negative and significant at the 5% level.

The results of the R-squared determination coefficient (0.5788) are consistent, which represents higher variance between the one-part models in the cloglog specification. The results of the R-squared determination coefficient in the first component of the two-part model shows less consistency with the other estimations (lower R-squared), showing a lower explanatory power for analyzing the probability of one European country to be on the eco-efficiency frontier (0.1766 in logit and 0.1785 in cloglog, respectively). Considering the panel sample of the 27 European countries analyzed, this subsample of efficient countries is much lower than the sample of the inefficient countries, which could explain the lower R-squared obtained in the first component of the two-part models.

Moreover, the results of the determination coefficient R-squared in the second component of the two-part models show high consistency with the other estimations, with higher variance under the cloglog specification (0.6674). The subsample of efficient countries is much higher than the sample of inefficient countries, which could explain the lower R-squared obtained in the second component of the two-part models. However, this result for the R-squared shows a good explanatory power for analyzing the probability of one European country being on the eco-efficiency frontier.

Table 6 reports for each model the calculated average partial effects (APE) for each explanatory variable and the one-part and two-part fractional models, respectively. The partial effect is estimated for each covariate, which was computed as the mean of the partial effects for each European country in our sample (EU 27).

For the analysis of the results from the average partial effects, the logit and cloglog specifications are considered for the one-part model, and for the first component of the two-part model, the logit, loglog, and cloglog as it is more consensual, while for the second component of the two-part models the cloglog, as these reveal to be more consensual across the specifications tests, which were carried out. Interpretation is to be done over cloglog. Both GHG emissions, CO_2_/area, and N_2_O/area, have a statistical and significant negative effect on the DEA eco-efficiency score, with a value of 3.11% and 1.49% in the one-part model and 15.25% and 11.65% in the two-part models, respectively. Another important result attained from the APE is that it is revealed that using pollutants as explanatory variables evidence a decreasing impact on the eco-efficiency values, as measured by the DEA score, simultaneously in the one-part and the two-part models considered. For instance, the PM2.5/area, PM10/area, and SOx/area decrease the DEA score by 3.48%, 3.68%, and 1.11% for the one-part model, and it contributes to bringing the inefficient countries closer to the eco-efficiency frontier, with an APE of 19.61%, 24.61% and 4.51% in the two-part models, respectively.

## 5. Results Discussion

**Figure 1:**
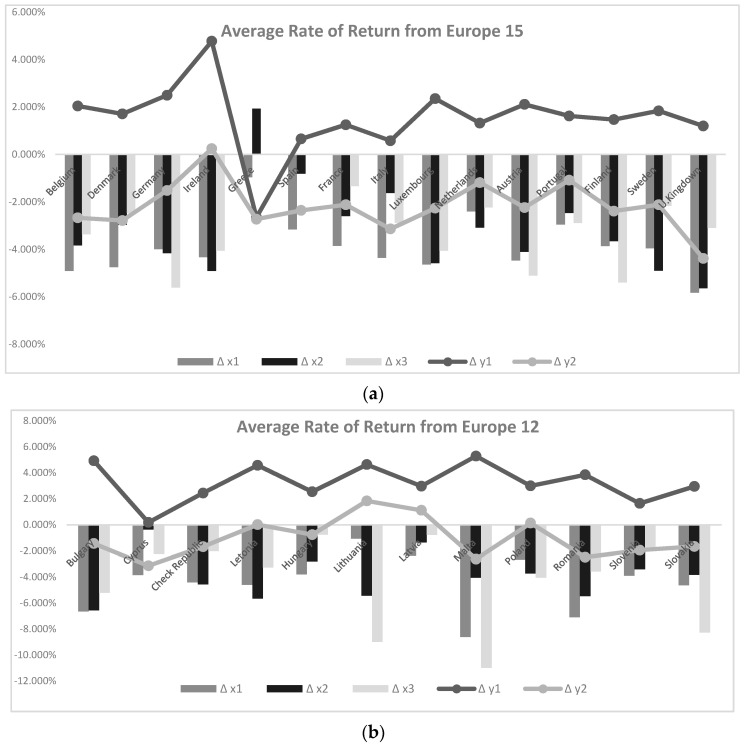
Evolution of the profitability rate of greenhouse gas emissions (GHG) emissions (x1, x2, x3 stand for CO_2_, CH_4_, N_2_O, respectively) in opposition to the evolution of the rate of return of the indicator of economic growth per capita (y1) and the rate of return of GHG (y2) for each country: (**a**) average rate of return from EU 15; (**b**) average rate of return from EU 12 (2008–2018 period).

**Figure 2:**
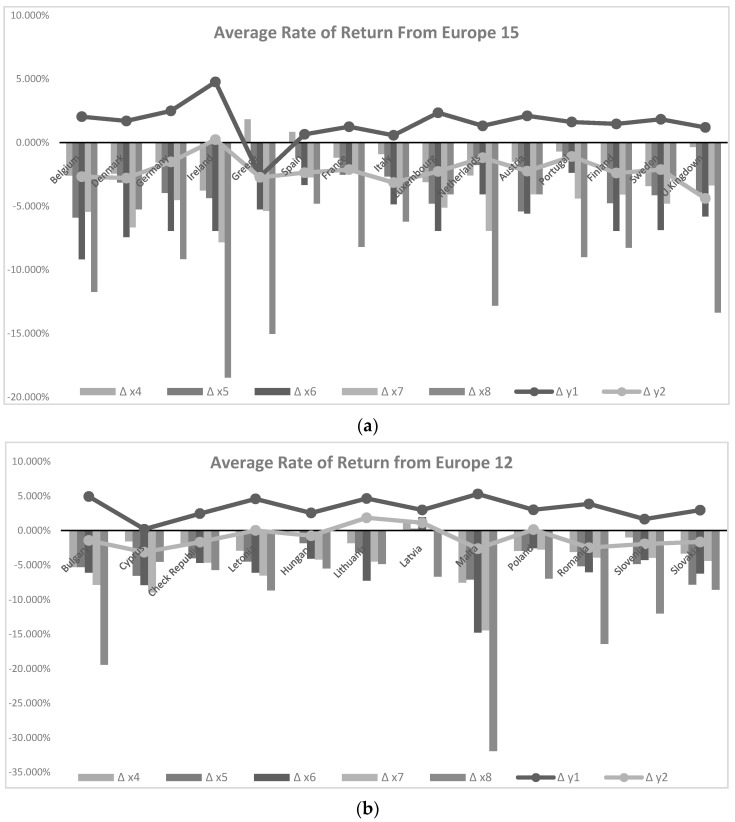
Evolution of the profitability rate of polluting gases (x4, x5, x6, x7, x8 stand for ammonia (NH_3_), nonmethane volatile organic compounds (NMVOC), particles with a diameter of less than 2.5 µm (PM2.5), particles with a diameter of less than 10 µm (PM10), SO_X_, respectively) in opposition to the evolution of the rate of return of the indicator of economic growth per capita (y1) and the rate of return of GHG (y2) for each country: (**a**) average rate of return from EU 15; (**b**) average rate of return from EU 12 (2008–2018 period).

Based on the results already found and presented in the previous section, we will, on one hand, sustain the statistical significance found for the drivers of the pollutants that influence the positioning in terms of eco-efficiency, specifically, based on an analysis of the evolution of the profitability rate of GHG (CO_2_, CH_4_, N_2_O), by area (Figure 1), and polluting gases (NH_3_, NMVOC, PM2.5, PM10, SO_X_), by area (Figure 2). This confrontation will be analyzed in opposition to the evolution of the rate of return of the indicator of economic growth per capita and the rate of return of GHG for each country, given that these two metrics were used as an output of eco-efficiency. On the other hand, taking into account the different timings of the accession of the different European economies to the European community, we break down this analysis into two distinct groups called EU 15 and EU 12, old and new, entering, Europe groups, and also taking into account the position of countries in the ranking of eco-efficiency found in the first stage of this article (DEA scores) (see Figure 1 and Figure 2).

Graphically, it is possible to verify that the country with the highest rates of change in profitability (in absolute terms) for GHGs and polluting gases in Ireland. This means that, from 2008 to 2018, this was the country that most mitigated its emissions, with particular emphasis on the emissions of sulfur dioxide (SOX: −18.46%) and particles with a diameter of less than 10 µm (PM10: −7.82%). Such reduction in emissions is accompanied by a greater increase in the return rate of the eco-efficiency indicator (0.40%). Other countries like Belgium, Denmark, Germany, Luxembourg, and the United Kingdom also perform well (eco-efficiency return rates of 0.31%, 0.28%, 0.27%, 0.33%, 0.28%, respectively).

Another analysis resulting from these two graphs in Figure 2 is, similarly to the previous Figure 1, on the rate of profitability of the polluting gas SO_X_, which was the best for Belgium (−11.74%), Ireland (−18.46%), Spain (−4.78%), the Netherlands (−12.81%) and the United Kingdom (−14.14%). The worst performance is provided by Greece and Spain, with the lowest reductions in emissions. Furthermore, Greece even registered an increase of these for methane (CH_4_:1.93%) and ammonia (NH_3_:1.84%), while Spain registered only an increase for the last polluting gas mentioned (0.85%) (translating into positive rates of return).

As observed from our results, both emissions and pollutants urgently need to be reduced overall in the 27 EU countries if the goal is to achieve the desired eco-efficiency, namely higher economic growth, but at the expense of lower pollution. Therefore, and carefully, policymakers should weigh the costs of environmental degradation and the benefits of increasing economic growth. To attain eco-efficiency, both policymakers and firms should jointly optimize the environment and the economy [34]. Eco-efficiency scores and improvement targets provide valuable insights into how EU countries contribute towards national wealth and affect economic growth, aiding policymakers in developing more effective regulations. Still, more research is required for policymakers to understand how this information could be transposed into national and overall EU regulations aiming to ensure sustainable development. This would help in a deeper understanding of how impacts are generated in EU countries and how individual countries could react to improve worldwide eco-efficiency keeping the sustainable development required on track.

Fostering renewable energy development efficiently and decommissioning fossil fuel generation gradually enhances EU countries’ eco-efficiency potential [37]. This would simultaneously reduce emissions and stimulate the growth of value-added. Resource management has been pointed as one of the main drivers of inefficiency in Europe [33], whereas the technological gap identified is mainly due to significant losses of human capital. Therefore, eco-efficiency improvements can only be reached through efficient use of productive factors, revealing the ongoing challenge placed to EU 27 countries of finding the correct balance between economic growth and environmental protection, that as our results indicate, would depend on the environmental pressure variable considered. Moreover, European countries, which include in their waste management a higher variety of waste, are more efficient than those relying upon landfilling [39], leading us to think in the urgent need of reformulating some of the current environmental policies.

GHG derived from productive processes strongly impacts the environment, driving a permanent climate change problem and global warming [30]. In ref. [48], the effects of eco-efficiency are explored on firm performance considering SMEs from 28 EU countries. Conclusions indicate that not all eco-strategies are positively related to performance in the short-run, suggesting a higher need for policy interventions. Therefore, increased production that could lead to economic growth is not always beneficial in the production sector leading to higher expenses and a higher weighting by firms of the environmental improvements that could be achieved. This demands stricter policies, but as well to benefits to be offered to the production sector to lead them to surpass higher expenses difficulties and to implement these policies, the only backup possible to face financial restrictions.

## 6. Conclusions

Considering concerns in raising the economic output of European countries while simultaneously preventing environmental concern has been at the forefront of the European agenda and Sustainable Development Goals. In other words, there is still a need to infer if countries are Eco-efficient and how they are evolving through time in this sense. With this in mind, this paper evaluates the evolution of eco-efficiency for the 27 European Union (EU) countries over the period 2008–2018. In this regard, we conducted a two-stage analysis, which computes eco-efficiency scores in the first stage for each of the pairs EU 27-year, through the nonparametric method data envelopment analysis (DEA), considering the ratio GDP per capita and greenhouse gas emissions (GHG) as the output. Technical efficiency scores were computed considering this ratio as the output and common variables reported in the literature as inputs. In the second stage, scores were used as a dependent variable in the proposed fractional regression model (FRM), whose determinants considered were in total eight pollutants (three greenhouse gases and five atmospheric pollutants).

Our results allowed us to observe that despite the EU has triggered several initiatives and regulations regarding environmental protection over the years; a lot more remains to be done. Namely, CO_2_/area and N_2_O/area effects are negative and significant, decreasing the eco-efficiency of the EU 27 countries. A separate analysis considering the EU 15 and the EU 12 countries is also presented. In fact, and under the used specifications in the FRM analysis presented, all pollutants in general increase the environmental burden for each EU 27 country, imposing the need for more interventions since results demonstrate their clear and negative influence over eco-efficiency. Eco-efficiency scores are higher under the variable returns to scale DEA model, and the overall significance of FRM models differs following the procedure adopted for the econometric part. The country with the lowest GHG emissions and pollutant gases was Ireland, being the country within the considered period that mostly reduced emissions, particularly SOx and PM10, increasing its score. For the future, we propose the inclusion of more independent variables into the analysis of the factors able to influence the countries individual eco-efficiency score under the FRM approach, as the percentage of fossil fuels and renewables in the total energy consumption and production of each country, as well, as trade and globalization variables, which certainly affect the binomial economic growth and environmental protection.

Results also pointed to different stages of the eco-efficiency process in EU countries. It is possible to verify that the country with the highest rates of change in profitability for GHGs and polluting gases in Ireland, thus the one that most mitigated its emissions, with particular emphasis on the emissions of sulfur dioxide and particles (PM10). Such reduction in emissions was accompanied by a greater increase in the return rate of the eco-efficiency indicator. Belgium, Denmark, Germany, Luxembourg, and the United Kingdom also perform well but are still in need of reducing more emissions. The worst performance is provided by Greece and Spain, with the lowest reductions in emissions. Greece even registered an increase of these for methane (CH_4_) and ammonia (NH_3_), while Spain registered only an increase for the last polluting gas mentioned. Another important result attained from the average partial effects is that using pollutants as explanatory variables evidence a decreasing impact on the eco-efficiency values, as measured by the DEA score (PM2.5/area, PM10/area, and SOx/area decrease the DEA score). Therefore, these contribute to bringing the inefficient countries closer to the eco-efficiency frontier. This also means that a greater emphasis should be placed on the reduction of gases to achieve environmental efficiency goals in EU countries, whereas the efficiency is greater the lower the number of pollutants. Still, both emissions and pollutants urgently need to be reduced overall in the 27 EU countries if the goal is to achieve the desired eco-efficiency, namely higher economic growth, but at the expense of lower pollution. Since gases and pollutants are a result of different emissions sources, policymakers should be aware that policies to mitigate GHG should be done in a heterogeneous way, not only considering the country but as well considering the emission source, as our results seem to indicate. Therefore, and carefully, policymakers should weigh the costs of environmental degradation and the benefits of increasing economic growth, heterogeneously and not by simply imposing general rules to be followed at the EU level.

In terms of future research, this analysis has limitations by not including the production process that involves several GHG emissions and that a given GDP can be obtained with different combinations of GHG. Thus, taking into account these limitations, it will be necessary to develop new works in an attempt to overcome these same limitations, either using nonparametric methods or nonparametric methods, in order to better understand and reinforce the explanation of the difference between eco-efficiency and concepts, such as sustainability, environmental effectiveness, or environmental performance indicators, even if the differences between them are considered minimal.

## Figures and Tables

**Table 1:** Variables descriptions, descriptive statistics, and sources: 2008–2018 period, for 1st stage (**a**) and for second stage (**b**).

(a) 1st Stage: DEA Method							
Variable	Description	Obs.	Mean	Std. Dev.	Min	Max	Source
Output							
GDP pc/(GHG/area)	The ratio of the value of gross domestic product per capita and the value of the volume of the GHG emissions by area of a given European country	297	23.43479	1.329979	20.45615	25.86164	Eurostat
Inputs							
GFCF per capita	Gross fixed capital formation (formerly gross domestic fixed investment)	297	−0.41147	0.47855	−1.6464	0.57486	World Bank
Labor per capita	The Labor force comprises people aged 15 and older, who supply labor for production during a specified period	297	−0.71090	0.072017	−0.88623	−0.61744	World Bank
Energy use/area	Energy use refers to the use of primary energy before transformation to other end-use fuels	297	0.37417	1.22472	0.004871	6.53248	World Bank
Electricity/area	The inputs used to generate electricity included oil, gas, coal, and derived fuels. Peat is also included in this category	297	−8.08690	2.08304	−12.9726	−3.35616	World Bank
Deviations temp	The indicator measures the development of deviations in average near-surface temperature for Europe	297	1.62	0.30451	1.06	2.11	Eurostat
**(b) 2nd Stage: Fractional Regression Models (FRM)**							
**Variable**	**Description**	**Obs.**	**Mean**	**Std. Dev.**	**Min**	**Max**	**Source**
CO2/area	Volume of CO_2_ emissions by area	297	−13.215	1.4682	−15.448	−9.3560	Eurostat
CH4/area	Volume of methane emissions by area	297	−18.4165	1.39757	−20.7865	−15.1238	Eurostat
N2O/area	Volume of nitrous oxide emissions by area	297	−21.4825	1.34095	−23.144	−18.2737	Eurostat
NH3/area	Volume of ammonia air pollutant emissions by area	297	−19.9800	1.36434	−22.0392	−16.5997	Eurostat
NMVOCs/area	Volume of nonmethane volatile organic compounds air pollutant emissions by area	297	−19.5869	1.35265	−21.3546	−16.0401	Eurostat
PM2.5/area	Volume of PM2.5 air pollutant by area	297	−21.5477	1.47132	−23.3426	−17.9860	Eurostat
PM10/area	Volume of PM10 air pollutant emissions by area	297	−20.8983	1.396479	−22.6495	−17.7629	Eurostat
SOx/area	Volume of total emissions of sulfur oxides (SOx) by area	297	−20.2309	1.529419	−22.6495	−16.2307	Eurostat

**Table 2:** Scores of technical eco-efficiency estimation in EU-27 under data envelopment analysis (DEA)-constant returns to scale (CRS): 2008–2018 period.

	2008	2009	2010	2011	2012	2013	2014	2015	2016	2017	2018	Average
Austria	0.6471	0.6625	0.9212	0.6226	0.7327	0.7396	0.4675	0.5401	0.5359	0.5637	0.4986	0.5848
Belgium	0.6164	0.6460	0.8973	0.6093	0.7163	0.7236	0.4577	0.5286	0.5244	0.5531	0.4878	0.5702
Bulgaria	0.6518	0.6722	0.9320	0.6323	0.7455	0.7552	0.4763	0.5511	0.5501	0.5814	0.5171	0.5953
Cyprus	0.6638	0.6770	0.9434	0.6401	0.7512	0.7549	0.4729	0.5474	0.5428	0.5723	0.5066	0.5969
Czech Republic	0.7051	0.7163	0.9974	0.6770	0.7933	0.7992	0.5036	0.5847	0.5802	0.6152	0.5453	0.6338
Denmark	0.6402	0.6527	0.9100	0.6159	0.7255	0.7328	0.4642	0.5363	0.5311	0.4936	0.6997	0.5729
Estonia	0.6641	0.6726	0.9258	0.6339	0.7491	0.7558	0.4802	0.5602	0.5539	0.5859	0.5210	0.5983
Finland	0.6756	0.6879	0.9538	0.6472	0.7623	0.7694	0.4860	0.5634	0.5562	0.5874	0.5178	0.6081
France	0.7020	0.7180	1.0000	0.6748	0.7925	0.7995	0.5040	0.5825	0.5767	0.6068	0.5353	0.6324
Germany	0.6997	0.7142	0.9931	0.6736	0.7901	0.7972	0.5047	0.5833	0.5784	0.6100	0.5389	0.6313
Greece	0.6506	0.6652	0.9191	0.6162	0.7172	0.7222	0.4556	0.5264	0.5212	0.5472	0.4824	0.5764
Hungary	0.7093	0.7180	1.0000	0.6771	0.7941	0.8033	0.5077	0.5873	0.5832	0.6171	0.5467	0.6361
Ireland	0.6659	0.6764	0.9311	0.6310	0.7398	0.7482	0.4744	0.5603	0.5542	0.5863	0.5196	0.5971
Italy	0.6764	0.6978	1.0000	0.6530	0.7816	0.7979	0.4892	0.5687	0.5621	0.5919	0.5171	0.6199
Latvia	0.6853	0.6867	0.9646	0.6467	0.7657	0.7747	0.4902	0.5665	0.5622	0.5945	0.5258	0.6125
Lithuania	0.6859	0.6949	0.9659	0.6581	0.7765	0.7876	0.4973	0.5746	0.5696	0.6015	0.5331	0.6193
Luxembourg	0.5737	0.5831	0.8193	0.5556	0.6514	0.6588	0.4173	0.4807	0.4778	0.5033	0.4449	0.5201
Malta	0.5149	0.5252	1.0000	0.4956	0.5840	0.5963	0.3796	0.4470	0.4463	0.4685	0.4185	0.4961
Netherlands	0.6243	0.6367	0.8861	0.5985	0.7040	0.7105	0.4473	0.5147	0.5105	0.5386	0.4762	0.5610
Poland	0.7069	0.7139	1.0000	0.6778	0.7969	0.8043	0.5095	0.5898	0.5823	0.6157	0.5445	0.6361
Portugal	0.6424	0.6648	1.0000	0.6319	0.8403	1.0000	0.4630	0.5417	0.5357	0.5606	0.4878	0.6255
Romania	0.6919	0.7022	1.0000	0.6594	0.7749	0.7897	0.5000	0.5796	0.5776	0.6132	0.5439	0.6262
Slovakia	0.6690	0.6841	0.9541	0.6469	0.7632	0.7700	0.4874	0.5639	0.5583	0.5884	0.5203	0.6078
Slovenia	0.6646	0.6778	0.9401	0.6356	0.7443	0.7520	0.4777	0.5520	0.5471	0.5779	0.5113	0.5972
Spain	0.7042	0.7201	1.0000	0.6741	0.7879	0.7962	0.5020	0.5800	0.5766	0.6068	0.5361	0.6316
Sweden	0.7046	0.7142	1.0000	0.6819	0.8028	0.8114	0.5113	0.5911	0.5852	0.6157	0.5406	0.6380
United Kingdom	0.6786	0.6978	1.0000	0.6564	0.7832	0.7878	0.4878	0.5699	0.5610	0.5890	0.5193	0.6192
Average (27 EU)	0.6635	0.6770	0.9576	0.6379	0.7543	0.7681	0.4783	0.5545	0.5497	0.5772	0.5199	0.6016

**Table 3:** Scores of technical eco-efficiency estimation in EU-27 under DEA-variable returns to scale (VRS): 2008–2018 period.

	2008	2009	2010	2011	2012	2013	2014	2015	2016	2017	2018	Average
Austria	0.9025	0.9090	0.9212	0.9101	0.9149	0.9162	0.9187	0.9204	0.9222	0.9212	0.9248	0.9165
Belgium	0.8912	0.9818	0.8973	0.9967	1.0000	0.9973	0.9886	0.9922	1.0000	1.0000	0.9968	0.9765
Bulgaria	0.9275	0.9774	0.9320	0.9826	0.9930	1.0000	0.9752	0.9824	1.0000	1.0000	1.0000	0.9791
Cyprus	0.9250	0.9255	0.9434	0.9314	0.9404	0.9394	0.9350	0.9385	0.9223	0.9273	0.9267	0.9323
Czech Republic	0.9826	0.9792	0.9974	0.9850	0.9930	0.9933	0.9719	0.9833	0.9858	0.9969	0.9975	0.9878
Denmark	0.8987	0.8984	0.9100	0.9037	0.9124	0.9149	0.9101	0.9142	0.9138	0.9166	0.9831	0.9160
Estonia	0.9254	0.9195	0.9258	0.9223	0.9378	0.9394	0.9266	0.9420	0.9411	0.9493	0.9530	0.9347
Finland	0.9409	0.9438	0.9538	0.9463	0.9527	0.9573	0.9592	0.9641	0.9588	0.9617	0.9622	0.9546
France	0.9892	0.9937	1.0000	0.9945	0.9989	1.0000	1.0000	1.0000	1.0000	1.0000	1.0000	0.9978
Germany	0.9831	0.9836	0.9931	0.6736	0.9937	0.9952	0.9892	0.9936	0.9954	1.0000	1.0000	0.9637
Greece	0.9957	0.9918	0.9191	0.9973	0.9970	1.0000	1.0000	1.0000	0.9969	0.9960	1.0000	0.9903
Hungary	1.0000	1.0000	1.0000	1.0000	1.0000	1.0000	0.9959	0.9960	1.0000	1.0000	1.0000	0.9993
Ireland	0.9347	0.9319	0.9311	0.9374	0.9508	0.9528	0.9436	0.9721	0.9674	0.9771	0.9738	0.9521
Italy	0.9847	0.9944	1.0000	1.0000	0.9931	1.0000	1.0000	1.0000	0.9993	0.9983	0.9963	0.9969
Latvia	0.9560	0.9398	0.9649	0.9423	0.9588	0.9631	0.9496	0.9552	0.9580	0.9655	0.9655	0.9562
Lithuania	0.9558	1.0000	0.9659	0.9740	0.9852	0.9834	0.9765	0.9744	0.9743	0.9767	0.9805	0.9770
Luxembourg	0.8135	0.8047	0.8193	0.8176	0.8195	0.8219	0.8178	0.8166	0.8219	0.8236	0.8255	0.8184
Malta	1.0000	0.9768	1.0000	0.8573	0.8183	0.7623	0.7362	0.7553	0.7612	0.7617	0.7685	0.8361
Netherlands	0.8691	0.8719	0.8861	0.8763	0.8850	0.8877	0.8860	0.8748	0.8804	0.8826	0.8859	0.8805
Poland	0.9994	0.9812	1.0000	0.9886	0.9982	1.0000	0.9875	0.9946	0.9925	1.0000	1.0000	0.9947
Portugal	0.9201	0.9362	1.0000	0.9583	0.9855	1.0000	0.9959	0.9874	0.9886	0.9709	0.9654	0.9735
Romania	0.9938	1.0000	1.0000	0.9842	0.9875	1.0000	0.9702	0.9863	1.0000	1.0000	1.0000	0.9929
Slovakia	0.9333	0.9361	0.9541	0.9425	0.9557	0.9573	0.9443	0.9509	0.9512	0.9556	0.9554	0.9488
Slovenia	0.9271	0.9275	0.9401	0.9260	0.9320	0.9349	0.9255	0.9309	0.9324	0.9385	0.9389	0.9322
Spain	0.9907	0.9930	1.0000	0.9933	0.9957	1.0000	1.0000	0.9967	1.0000	1.0000	1.0000	0.9972
Sweden	0.9778	0.9757	1.0000	0.9913	0.9972	1.0000	1.0000	1.0000	1.0000	1.0000	0.9977	0.9945
United Kingdom	0.9677	0.9736	1.0000	0.9819	0.9904	0.9887	0.9872	0.9955	0.9887	0.9870	0.9899	0.9864
Average (27 EU)	0.9476	0.9536	0.9576	0.9413	0.9588	0.9594	0.9515	0.9562	0.9575	0.9595	0.9625	0.9550

**Table 4:** Specification tests for the one-part model and two-part models (in its first and second components): 2008–2018 period.

	One-Part Models				Two-Part Models							
					First Component				Second Component			
	Logit	Probit	Loglog	Cloglog	Logit	Probit	Loglog	Cloglog	Logit	Probit	Loglog	Cloglog
RESET test	17.59 ***	12.74 ***	19.09 ***	3.87	3.53 *	2.506	1.546	2.64	6.340 **	4.076 **	6.93 ***	1.760
GOFF-I test	14.86 ***	12.68 ***		2.82	1.939	1.547		0.678	6.279 **	4.056 **		1.744
GOFF-II test	16.76 ***	12.64 ***	17.79 ***		0.475	2.686 *	1.640		6.340 **	4.136 **	7.01 ***	
GGOFF	17.82 ***	12.68 ***	17.79 ***	2.82	10.29 ***	8.75 **	1.640	0.678	6.349 **	4.241	7.01 ***	1.744
	***P*-test**											
H1: FRM II–logit		11.76 ***	15.92 ***	3.85		9.37 ***	8.06 ***	0.035		3.490 **	7.12 ***	1.307
H1: FRM II–probit	19.00 ***		20.49 ***	3.48	3.368 *		4.135 **	0.500	6.98 ***		8.00 ***	1.580
H1: FRM II-loglog	14.40 ***	11.67 ***		3.89	0.000	0.273		0.038	6.102 **	3.543 **		1.338
H1: FRM II-cloglog	20.06 ***	13.46 ***	21.82 ***		0.547	4.623 **	6.49 ***		7.66 ***	4.459 **	8.69 ***	

Note: ***, ** and * denote test statistical significance at 1%, 5% and 10%, respectively. FRM—fractional regression model. GOFF—generalized goodness-of-functional form test for binary and fractional regression models.

**Table 5:** Estimation results for the fractional regression model (27 European countries sample): 2008–2018 period.

	One-Part Models		Two-Part Models			
			First Component		Second Component	
	Logit	Probit	Logit	Probit	Logit	Probit
	DEA-VRS	DEA-VRS	DEA-VRS	DEA-VRS	DEA-VRS	DEA-VRS
CO2/area	−0.64088 ***	−0.33261 ***	−0.95707 **	−0.40055	−0.66960 ***	−0.34815 ***
CH4/area	−0.34683*	−0.14423	−0.82603	−0.52906 *	−0.62155 ***	−0.26387 ***
N_2_0/area	−0.44305 ***	−0.19295 ***	−0.99340 **	−0.62872 **	−0.27661 **	−0.11634 **
NH3/area	−0.09582	−0.02787	−1.86841 ***	1.21054 ***	−0.40126 *	−0.18192 *
NMVOC/area	−0.06458	−0.02407	−0.23026	−0.23663	−0.10471	−0.08939
PM2.5/area	−0.96719 ***	−0.42848 ***	−1.46954 ***	−0.80893 ***	−0.64478 ***	−0.29285 ***
PM10/area	−1.08304 ***	−0.46655 ***	−1.75844 ***	−0.93809 **	−0.76547 ***	−0.33144 ***
SOx/area	−0.25321 ***	−0.12536 ***	−0.33874	−0.19504 *	−0.20275 ***	−0.10602 ***
*Cons*	−0.51518	−0.10730	−5.82937	−3.68620	0.18205	0.44460
*Obs*	297	297	297	297	235	235
*R* ^2^	0.5673	0.5737	0.1766	0.1660	0.6566	0.6627
	**One-Part Models**		**Two-Part Models**			
			**First Component**		**Second Component**	
	**Loglog**	**Cloglog**	**Loglog**	**Cloglog**	**Loglog**	**Cloglog**
	DEA-VRS	DEA-VRS	DEA-VRS	DEA-VRS	DEA-VRS	DEA-VRS
CO2/area	−0.59078 ***	−0.25523 ***	−0.22788	−0.91315 **	−0.61980 ***	−0.26839 ***
CH4/area	−0.34912*	−0.08725	−0.51877 *	−0.65527	−0.61959 ***	−0.16357 ***
N_2_0/area	−0.44391 ***	−0.12184 ***	−0.63246 **	−0.71592 *	−0.27993 **	−0.06893 **
NH3/area	−0.09551	−0.00817	−1.20376 ***	−1.45851 ***	−0.39595 *	−0.12218 **
NMVOC/area	−0.09565	−0.05323	−0.30238	−0.19500	−0.07657	−0.09140 **
PM2.5/area	−0.94199 ***	−0.28483 ***	−0.73603 ***	−1.19409 ***	−0.62996 ***	−0.19917 ***
PM10/area	1.06996 ***	−0.30091 ***	−0.82145 **	−1.50290 ***	−0.75967 ***	−0.21238 ***
SOx/area	−0.23543 ***	−0.09063 ***	−0.17962 *	0.26956	−0.18646 ***	−0.08114 ***
*Cons*	−0.51518	−0.08614	−3.50904	−4.59116	−0.16780	−0.33439
*Obs*	297	297	297	297	235	235
*R* ^2^	0.5634	0.5788	0.1530	0.1785	0.6527	0.6674

Notes: *, **, and *** mean statistically significance at 10%, 5%, and 1%, respectively. Cons—constant; Obs—observations.

**Table 6:** Sample average of partial effects (APE): 2008–2018 period.

	One-Part Model		Two-Part Model	
			Logit (1st Component)	
	logit	cloglog	loglog	cloglog
CO2/area	−0.02582	−0.03118	−0.15070	−0.15251
CH4/area	−0.01397	−0.01066	−0.10093	−0.10179
N_2_0/area	−0.01785	−0.01488	−0.11675	−0.11653
NH3/area	−0.00386	−0.00100	−0.23131	−0.23177
NMVOC/area	−0.00260	−0.00650	−0.03076	−0.02938
PM2.5/area	−0.03897	−0.03479	−0.19584	−0.19617
PM10/area	−0.04364	−0.03676	−0.24626	−0.24609
SOx/area	−0.01020	−0.01107	−0.04451	−0.04506

Note: logit (1st Component) + loglog and logit (1st Component) + cloglog.

## Data Availability

Data were collected from publicly archived datasets analyzed or generated during the study and presented in Table 1a,b.

## Appendix A

**Table A1:** Correlation matrix for inputs and outputs in DEA eco-efficiency ratios computed: 2008–2018 period. See Table 1a for data details.

	GDP pc/GHG/Area	GFCF per Capita	Labor per Capita	Energy Use/Area	Electricity/Area	Deviations Temp
GDP pc/GHG/area	1.0000					
GFCF per capita	0.3190	1.0000				
Labor per capita	−0.2070	0.5952	1.0000			
Energy use/area	−0.1753	0.1049	0.2268	1.0000		
Electricity/area	−0.4173	0.5803	0.7482	0.5269	1.0000	
Deviations temp	0.4637	0.8552	0.5441	0.1974	0.5608	1.0000
